# The Impact of Severe Wildfires on Mental Health: Prevalence of Major Depressive Disorder and Related Factors among Residents in Alberta and Nova Scotia, Canada.

**DOI:** 10.1192/j.eurpsy.2024.538

**Published:** 2024-08-27

**Authors:** W. Mao, R. Shalaby, B. Agyapong, G. Obuobi-Donkor, R. Dias, V. I. Agyapong

**Affiliations:** ^1^Psychiatry, University of Alberta, Edmonton; ^2^Psychiatry, Dalhousie University, Halifax, Canada

## Abstract

**Introduction:**

Hundreds of fires have been burning from coast to coast across the country since March 2023, putting Canada on track to experience the worst wildfire season ever. From East to West, provinces such as Quebec, Ontario, Nova Scotia, Alberta, and British Columbia have been particularly affected by large and uncontrollable wildfires.

**Objectives:**

This study aimed to determine the prevalence and predictors of depression symptoms among residents of Alberta and Nova Scotia during the Canadian wildfires of 2023.

**Methods:**

This study conducted a cross-sectional quantitative survey for data collection. In the period between 14th May and 23rd June 2023, an online survey was administered using REDCap. Through the Text4Hope program, participants subscribe to receive supportive SMS messages daily. After the first message, participants were invited to complete an online questionnaire, containing demographic information, wildfire-related information, and responses to the Patient Health Questionnaire-9 (PHQ-9) for depression assessment. SPSS version 25 was used to analyze the data. Descriptive, univariate, and multivariate regression analyses were employed.

**Results:**

A total of 298 respondents completed the online survey out of 1802 who accessed it, resulting in a response rate of 16.54 %. Most of the respondents were females (85.2%, 253), below 40 years of age (28.3%, 84), employed (63.6%, 189), and in a relationship (56.4%, 167). A historical depression diagnosis (OR = 3.15; 95% CI: 1.39–7.14) was a significant predictor of moderate to severe MDD in our study. While employment status did not significantly predict MDD, unemployed individuals were two times more likely to report moderate-to-severe symptoms of MDD than employed individuals (OR = 2.46; 95% CI: 1.06–5.67). Among the total sample population, the moderate to severe MDD prevalence was 50.4%, whereas it was 56.1% among those living in wildfire-affected areas.

**Image:**

**Figure:**
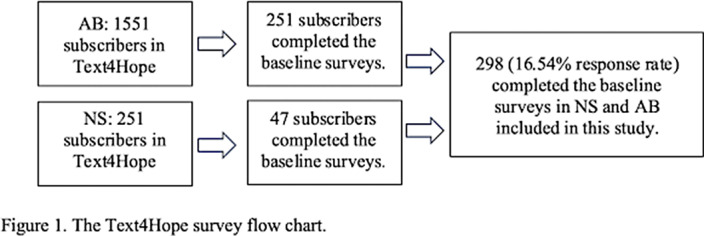

**Image 2:**

**Figure:**
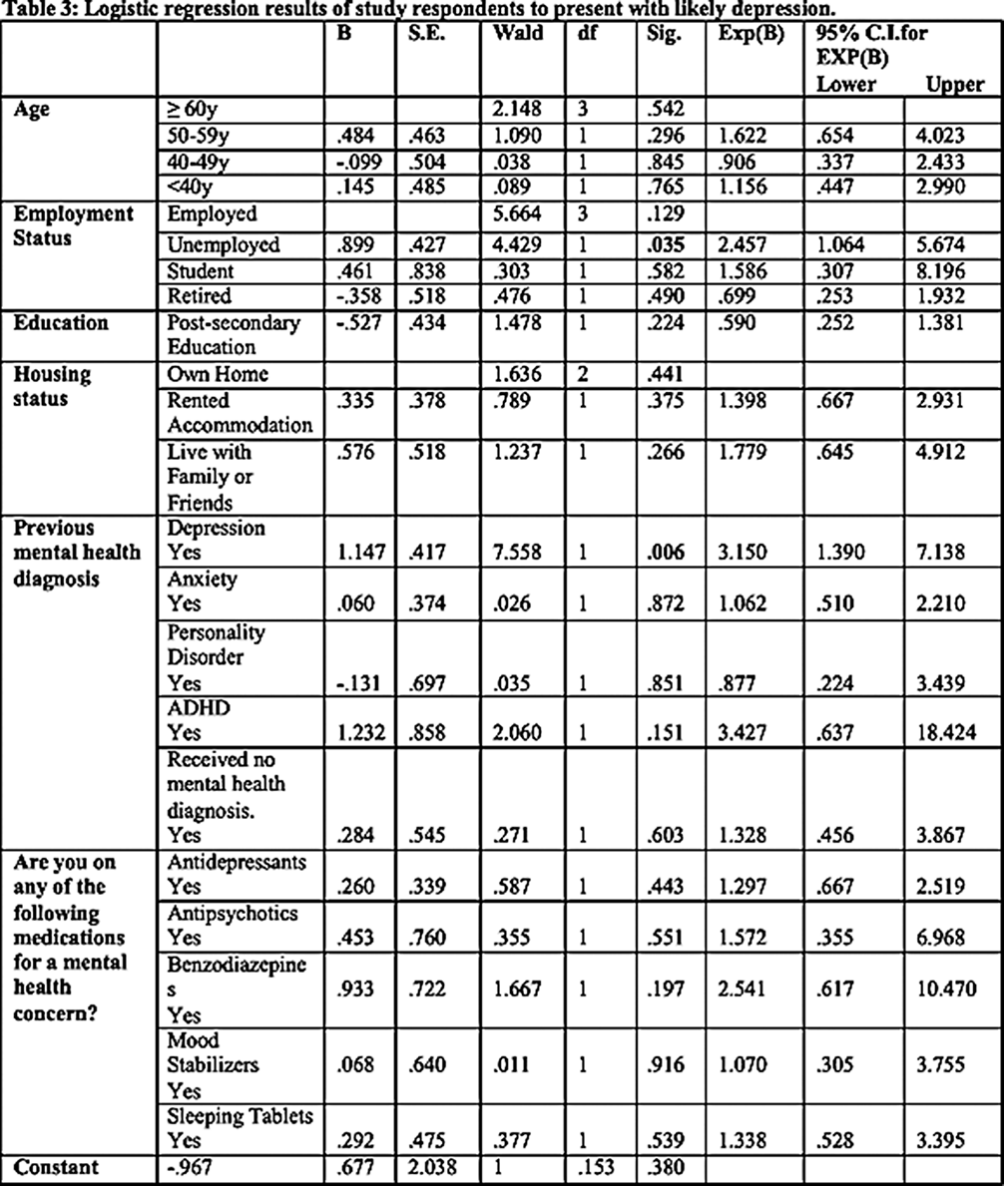

**Conclusions:**

As a result of our study, the development of moderate to severe MDD symptoms during wildfire disasters was significantly associated with a history of depression diagnosis. Although employment status did not significantly predict MDD, unemployed individuals had a greater likelihood of experiencing moderate-to-severe symptoms than employed individuals. Further research is necessary to ascertain reliable predictors of mental health issues among those who have experienced disasters, as well as to offer appropriate interventions and treatment options to the communities and individuals who are most vulnerable.

**Disclosure of Interest:**

None Declared

